# Special Issue “The Role of Oxidative Stress and Antioxidants in Human Disease”

**DOI:** 10.3390/ijms27208965

**Published:** 2026-10-09

**Authors:** Federica Mannino

**Affiliations:** Department of Medicine and Surgery, University of Enna “Kore”, Contrada Santa Panasia, 94100 Enna, Italy; federica.mannino@unikore.it

## 1. Introduction

Oxidative stress is widely recognized as a key mechanism involved in the onset and progression of numerous human diseases. Originally described as an imbalance between oxidants and antioxidants, the concept has evolved toward a broader framework encompassing the disruption of redox signaling and control [1,2,3]. Reactive oxygen species (ROS), including superoxide and hydrogen peroxide, arise from several cellular sources, with mitochondria and NADPH oxidases (NOXs) playing particularly important roles [4,5,6,7,8]. When produced in excess or insufficiently controlled by antioxidant defenses, ROS can induce oxidative modifications of lipids, proteins, and nucleic acids and contribute to mitochondrial dysfunction, inflammation, altered metabolism, cellular senescence, and cell death [2,4,5,6].

Recent advances in redox biology have substantially changed our understanding of these processes. ROS are no longer regarded solely as harmful metabolic by-products but also as physiological signaling molecules regulating proliferation, differentiation, metabolism, immune responses, and adaptation to cellular stress [2,3,4,6]. This dual role has led to the distinction between oxidative eustress, representing physiological redox signaling, and oxidative distress, characterized by excessive or dysregulated oxidant production [2,3]. At the same time, increasing attention has been directed toward reductive stress, in which excessive reducing capacity can disrupt mitochondrial metabolism, protein homeostasis, and redox-sensitive signaling [9]. Thus, current research increasingly views disease as the consequence of disturbed redox homeostasis, rather than simply excessive ROS production.

Despite these advances, major gaps remain. The precise contribution of oxidative stress to the initiation versus progression of many diseases is still unclear, reliable biomarkers capable of defining individual redox status are lacking, and the tissue- and compartment-specific effects of ROS remain difficult to assess. Moreover, despite compelling experimental evidence, antioxidant supplementation has frequently yielded inconsistent clinical outcomes—the so-called “antioxidant paradox” [10,11,12]. These limitations highlight the need to identify disease-specific redox mechanisms and to determine when, where, and in whom antioxidant or redox-modulating interventions may be beneficial.

Within this framework, the Special Issue “The Role of Oxidative Stress and Antioxidants in Human Disease” brings together clinical, preclinical, and mechanistic studies addressing redox imbalance across diverse pathological conditions, including HIV-associated renal and cardiovascular complications, myocardial ischemia–reperfusion injury, diabetes-associated cataract, adipocyte lipid accumulation, CDKL5 deficiency disorder, and human infertility. Collectively, these contributions help bridge mechanistic and translational perspectives and illustrate both the therapeutic potential and the limitations of interventions aimed at modifying redox homeostasis.

## 2. Overview of the Published Articles

A first important theme emerging from this Special Issue concerns the relationship between oxidative stress and chronic comorbidities in people living with HIV (PLWH). Despite the effectiveness of antiretroviral therapy (ART), persistent oxidative and inflammatory abnormalities may contribute to long-term organ dysfunction.

Choshi et al. [Contribution 1] investigated the relationship between oxidative stress markers and renal function in PLWH. The authors assessed malondialdehyde (MDA), superoxide dismutase (SOD), and total antioxidant capacity in relation to cystatin C-based estimated glomerular filtration rate. PLWH receiving ART showed higher MDA concentrations, while SOD activity was positively associated with renal function. These findings suggest that altered redox homeostasis may accompany early renal dysfunction and identify endogenous antioxidant capacity as a potentially relevant marker of renal health. However, the pilot and cross-sectional design leaves unresolved whether oxidative abnormalities precede renal impairment or arise as a consequence of it, highlighting the need for prospective longitudinal studies.

Mokoena et al. [Contribution 2] addressed a related knowledge gap by examining the role of myeloperoxidase (MPO) in oxidative stress and cardiovascular risk in PLWH. MPO provides a mechanistic connection between persistent inflammation, oxidative modification of lipoproteins, endothelial dysfunction, and atherosclerotic processes. The available evidence supports an association between sustained MPO activation and cardiovascular risk-related pathways, although the contribution of ART duration and other clinical variables remains incompletely defined. Together, these two contributions emphasize the need for integrated redox and inflammatory biomarkers capable of identifying PLWH at increased risk of chronic renal and cardiovascular complications.

Mitochondrial redox biology represents another major area of recent development. Rather than being viewed simply as sources of harmful ROS, mitochondria are increasingly recognized as dynamic regulators of bioenergetics, redox signaling, and cellular adaptation. Habimana et al. [Contribution 3] explored this concept in myocardial ischemia–reperfusion injury using complementary in vivo, ex vivo, and in vitro approaches. Low-dose endotoxin preconditioning preserved myocardial and mitochondrial function, reduced mitochondrial superoxide generation, and improved cellular bioenergetics during ischemic or hypoxic stress. These findings suggest that protection against oxidative injury may be achieved by preserving mitochondrial function and activating adaptive stress responses rather than simply scavenging ROS. Further research is required to identify the specific signaling pathways responsible for this protective phenotype and determine whether safer pharmacological approaches can reproduce such preconditioning in humans.

The study by Loi et al. [Contribution 4] extends the relevance of redox dysfunction to CDKL5 deficiency disorder (CDD). In cellular and animal models, Coenzyme Q10 (CoQ10) reduced ROS accumulation and oxidative damage and improved several indices of cellular homeostasis. This work provides evidence that oxidative abnormalities may contribute to the phenotype of a genetically determined neurodevelopmental disorder and identifies mitochondrial redox modulation as a potential therapeutic avenue. Nevertheless, the gap between preclinical efficacy and clinical benefit remains substantial, and future studies will need to establish appropriate dosing, treatment windows, biomarkers of response, and ultimately the efficacy and safety of this approach in patients.

The translational potential of antioxidant supplementation is also addressed by Hayashi et al. [Contribution 5], who investigated a lutein-containing supplement in diabetic patients with cataracts. The study revealed differences in ocular oxidative stress between diabetic and non-diabetic subjects and showed a reduction in carboxymethyl-lysine following supplementation in the diabetic group. These findings provide an intriguing connection between oxidative stress, glycation, and diabetic ocular complications. However, larger controlled studies are needed to determine the clinical relevance of these biochemical changes and to distinguish the specific contribution of lutein from that of other components of the supplement.

Natural products represent another potential source of redox-modulating compounds. Montaldo et al. [Contribution 6] demonstrated that an aqueous extract of *Peumus boldus* reduced intracellular ROS and lipid accumulation in differentiated 3T3-L1 adipocytes. These results support a mechanistic relationship between redox state and lipid metabolism and suggest that plant-derived compounds may provide useful leads for metabolic research. At the same time, the study highlights a recurring translational gap in antioxidant research: promising in vitro activity does not necessarily predict clinical efficacy. Characterization of active compounds, bioavailability, pharmacokinetics, safety, and efficacy in appropriate in vivo models will therefore be essential.

Finally, Moustakli et al. [Contribution 7] broaden the conceptual scope of the Special Issue by reviewing the impact of reductive stress on human infertility. Physiological ROS generation is essential for several reproductive processes, and excessive antioxidant activity may interfere with redox-dependent functions of gametes. This contribution is particularly relevant because it challenges the assumption that increasing antioxidant capacity is invariably beneficial. Instead, it reinforces the emerging view that both oxidative and reductive extremes can be detrimental and that therapeutic strategies should aim to restore an optimal redox state.

Taken together, these seven contributions address several important gaps in current redox research. They identify potential biomarkers of oxidative imbalance in human disease, examine the intersection between oxidative stress and inflammation, explore mitochondrial preservation as an alternative to non-specific ROS scavenging, evaluate antioxidant and natural-product interventions in clinical and experimental settings, and highlight reductive stress as an often-overlooked consequence of excessive antioxidant activity. Importantly, the methodological diversity of the Special Issue—from clinical observations and evidence synthesis to cellular and animal models—illustrates the need to integrate mechanistic and translational approaches in order to understand the context-dependent role of redox imbalance in disease.

## 3. Conclusions and Future Perspectives

The contributions to this Special Issue reflect a broader evolution in redox biology: the field is moving from a relatively simple model of oxidative damage and antioxidant protection toward a more nuanced understanding of redox homeostasis. ROS can be both damaging and physiologically essential, while antioxidants can be protective in one biological context but ineffective or potentially detrimental in another. The distinction between oxidative eustress, oxidative distress, and reductive stress therefore provides a more appropriate framework for understanding the complex role of redox processes in human disease. Several important challenges nevertheless remain. First, establishing causality is essential. Oxidative stress may initiate disease, amplify established pathology, or arise secondarily from tissue dysfunction, and these roles may vary according to disease stage. Longitudinal studies will therefore be required to determine whether redox biomarkers predict disease onset and progression rather than merely reflecting existing damage. Second, future studies should improve the specificity and standardization of redox biomarkers. Individual measurements such as MDA, SOD, MPO, glutathione status, or total antioxidant capacity provide valuable but incomplete information. Combining multiple redox markers with inflammatory, metabolic, and mitochondrial parameters may enable more accurate characterization of individual redox phenotypes. Third, greater attention should be paid to the spatial and temporal organization of redox signaling. The biological consequences of ROS depend on their molecular identity, concentration, cellular source, subcellular localization, and duration of exposure. Emerging approaches capable of monitoring redox changes in specific cellular compartments and in real time may substantially improve our understanding of disease-specific mechanisms. Fourth, translation of antioxidant interventions requires a shift toward precision redox medicine. Future clinical trials should consider baseline redox status, disease stage, dose, bioavailability, treatment duration, and concomitant therapies. Biomarker-guided patient stratification may help identify individuals most likely to benefit from a specific intervention while avoiding excessive antioxidant exposure and reductive stress. Finally, future research should increasingly integrate multi-omics technologies, advanced mitochondrial phenotyping, redox-sensitive imaging, and systems biology with well-designed longitudinal studies and randomized clinical trials. Such approaches may help identify redox signatures associated with particular disease phenotypes and reveal new molecular targets for selective intervention. Overall, this Special Issue highlights both the remarkable breadth and the complexity of redox biology in human disease. Its contributions support a transition from asking whether oxidative stress is present toward determining which redox pathways are altered, where and when these alterations occur, and whether they can be selectively corrected. Addressing these questions will be crucial for translating advances in redox biology into effective and individualized therapeutic strategies.
